# Outcomes after major surgery in patients with myasthenia gravis: A nationwide matched cohort study

**DOI:** 10.1371/journal.pone.0180433

**Published:** 2017-06-30

**Authors:** Yi-Wen Chang, Yi-Chun Chou, Chun-Chieh Yeh, Chaur-Jong Hu, Chih-Jen Hung, Chao-Shun Lin, Ta-Liang Chen, Chien-Chang Liao

**Affiliations:** 1 Department of Anesthesiology, Chia-Yi Branch, Taichung Veterans General Hospital, Chiayi, Taiwan; 2 Department of Anesthesiology, Taichung Veterans General Hospital, Taichung, Taiwan; 3 Department of Physical Medicine and Rehabilitation, China Medical University Hospital, Taichung, Taiwan; 4 Department of Surgery, China Medical University Hospital, Taichung, Taiwan; 5 Department of Surgery, University of Illinois, Chicago, Illinois, United States of America; 6 Department of Neurology, Shuang Ho Hospital, Taipei Medical University, New Taipei City, Taiwan; 7 Department of Anesthesiology, Taipei Medical University Hospital, Taipei, Taiwan; 8 Health Policy Research Center, Taipei Medical University Hospital, Taipei, Taiwan; 9 Department of Anesthesiology, School of Medicine, College of Medicine, Taipei Medical University, Taipei, Taiwan; 10 School of Chinese Medicine, College of Chinese Medicine, China Medical University, Taichung, Taiwan; 11 Department of Anesthesiology, Shuan Ho Hospital, Taipei Medical University, New Taipei City, Taiwan; Universitatsspital Basel, SWITZERLAND

## Abstract

**Objective:**

To validate the comprehensive features of adverse outcomes after surgery for patients with myasthenia gravis.

**Methods:**

Using reimbursement claims from Taiwan’s National Health Insurance Research Database, we analyzed 2290 patients who received major surgery between 2004 and 2010 and were diagnosed with myasthenia gravis preoperatively. Surgical patients without myasthenia gravis (n = 22,900) were randomly selected by matching procedure with propensity score for comparison. The adjusted odds ratios and 95% confidence intervals of postoperative adverse events associated with preoperative myasthenia gravis were calculated under the multiple logistic regressions.

**Results:**

Compared with surgical patients without myasthenia gravis, surgical patients with myasthenia gravis had higher risks of postoperative pneumonia (OR = 2.09; 95% CI: 1.65–2.65), septicemia (OR = 1.31; 95% CI: 1.05–1.64), postoperative bleeding (OR = 1.71; 95% CI: 1.07–2.72), and overall complications (OR = 1.70; 95% CI: 1.44–2.00). The ORs of postoperative adverse events for patients with myasthenia gravis who had symptomatic therapy, chronic immunotherapy, and short-term immunotherapy were 1.76 (95% CI 1.50–2.08), 1.70 (95% CI 1.36–2.11), and 4.36 (95% CI 2.11–9.04), respectively.

**Conclusions:**

Patients with myasthenia gravis had increased risks of postoperative adverse events, particularly those experiencing emergency care, hospitalization, and thymectomy for care of myasthenia gravis. Our findings suggest the urgency of revising protocols for perioperative care for these populations.

## Introduction

Myasthenia gravis, an autoimmune disorder, mainly presents as diplopia, ptosis, fluctuating muscle weakness, and even respiratory failure [1]. Global estimates note its incidence and prevalence between 1950 and 2007 were about 5 and 77.7 per 100,000 persons, and mortality was 0.1–0.9 per 100,000 persons [2]. Annual medical costs for myasthenia gravis in the United States were found to be as high as $15,675 per patient [3]. Mental disorders, limited physical activity, and poor quality of life are significant problems for this socially vulnerable population [4,5]. Thymectomy was considered as an invasive but definite treatment procedure for patients with myasthenia gravis [6–11]. With the increasing incidence of myasthenia gravis in elderly people due to longer life expectancy and improved diagnostic methods, myasthenia gravis patients may be receiving increasing numbers of surgeries other than thymectomy [12].

Previous studies found that patients with myasthenia gravis were more likely to have perioperative complications such as exacerbated muscle weakness, residual muscle paralysis, and cholinergic and myasthenia crises [7–13]. However, these studies were limited to single medical institutes [7–11], or focused on a specific surgical procedure [7–11], lacked a control group [8–10], had a small sample size [7,8] or inadequate adjustment for potential confounding effects [8–10]. Most available information from previous studies are focused on long-term prognosis of thymectomy or different thymectomy technique comparisons [9–11], while general features in this population undergoing regular surgical procedures were ignored [7–11].

Using Taiwan’s National Health Insurance Research Database, we conducted a population-based study to investigate adverse events after major surgeries in patients with myasthenia gravis compared with the general population.

## Methods

### Data sources

Research data were obtained from reimbursement claims of Taiwan’s National Health Insurance Program, which was implemented in March 1995 and covers more than 99% of the 22.6 million Taiwan residents. The National Health Research Institutes established the database to record all beneficiaries’ medical services for public research interest, including inpatient and outpatient demographics, primary and secondary diagnoses, procedures, prescriptions, and medical expenditures. The validity of this database has been favorably evaluated, and research articles based on it have been accepted in prominent scientific journals worldwide [14–17].

### Ethical approval

Insurance reimbursement claims from the National Health Insurance Research Database are available for public access. To protect personal privacy, the electronic database with patient identifications was decoded and scrambled for further research access. Informed consent was not required because of this privacy protection. However, this study was evaluated and approved by the institutional review board of Taipei Medical University (TMU-JIRB-201505055; TMU-JIRB-201404070) [14–17].

### Study design

We examined medical claims and identified 2290 patients aged ≥ 20 years with preoperative myasthenia gravis from 3,177,239 patients who underwent major inpatient major surgeries between 2004 and 2010 in Taiwan. These surgeries required general, epidural, or spinal anesthesia, and hospitalization for more than one day. To identify patients with myasthenia gravis strictly, we required at least two visits for medical services with a principal diagnosis of myasthenia gravis within the 24-month preoperative period. We matched each surgical patient with myasthenia gravis with 10 randomly selected surgical patients without myasthenia gravis by sex, age, operation in a teaching hospital or not, low-income status, mental disorders, hypertension, diabetes, chronic obstructive pulmonary disease, hyperlipidemia, liver cirrhosis, congestive heart failure, renal dialysis, and types of surgery and anesthesia, and conducted the analysis with propensity score matching procedure.

### Measures and definition

We identified income status by defining low-income patients as those qualifying for waived medical co-payment, because this status is verified by the Bureau of National Health Insurance. Also recorded were whether the surgery was performed in a teaching hospital and the types of surgery and anesthesia. We used the *International Classification of Diseases*, *Ninth Revision*, *Clinical Modification* (ICD-9-CM) to define preoperative coexisting medical conditions and postoperative complications. Preoperative myasthenia gravis (ICD-9-CM 358.0) was defined as the major exposure. Coexisting medical conditions were determined from medical claims for the 24-month preoperative period. These included mental disorders (ICD-9-CM 290–319), hypertension (ICD-9-CM 401–405), diabetes, (ICD-9-CM 250), chronic obstructive pulmonary disease (ICD-9-CM 490–496), hyperlipidemia (ICD-9-CM 272.0, 272.1, and 272.2), liver cirrhosis (ICD-9-CM 571), and renal dialysis (administration code D8, D9).

In-hospital 30-day mortality after the index surgery was considered the study’s primary outcome. Eight major surgical postoperative complications were analyzed as secondary outcomes after the index surgery, including pneumonia (ICD-9-CM 480–486), septicemia (ICD-9-CM 038 and 998.5), stroke (ICD-9-CM 430–438), postoperative bleeding (ICD-9-CM 998.0, 998.1 and 998.2), deep wound infection (ICD-9-CM 958.3), acute myocardial infarction (ICD-9-CM 410), pulmonary embolism (ICD-9-CM 415), and acute renal failure (ICD-9-CM 584). Prolonged hospital stay, intensive care unit stay, and in-hospital medical expenditures (including claims for prescriptions/medications, physician fees, ward fees, laboratory examinations, radiologic images, medical treatments. surgical procedures, and postoperative care) were also considered secondary outcomes. These outcomes were compared between patients with and without preoperative myasthenia gravis. Postoperative length of stay was categorized into quartiles, with prolonged stay defined as the highest quartile. Postoperative medical expenditure also was similarly categorized into quartiles. Types of treatment for patients with myasthenia gravis were also considered including symptomatic therapy, chronic immunotherapy, and short-term immunotherapy (such as plasmapheresis and intravenous immunoglobulin).

### Statistical analysis

We developed a non-parsimonious multivariable logistic regression model to estimate a propensity score for preoperative myasthenia gravis, irrespective of outcome. Clinical significance guided the initial choice of covariates in this model: sex, age, types of surgery and anesthesia, operation in teaching hospital or not, low-income status, mental disorders, hypertension, diabetes, chronic obstructive pulmonary disease, hyperlipidemia, liver cirrhosis, congestive heart failure, and renal dialysis. We used a structured iterative approach to refine this model with the goal of achieving covariate balance within the matched pairs. The chi-square tests were used to measure covariate balance and *P* < 0.05 was suggested to represent meaningful covariate imbalance. We matched patients with myasthenia gravis to patients without myasthenia gravis using a greedy-matching algorithm with a calliper width of 0.2 standard deviation of the log odds of the estimated propensity score. This method has been estimated to remove 98% of the bias from measured covariates [18,19].

Adjusted odds ratios (ORs) with 95% CIs for 30-day postoperative complications and mortality between patients with and without myasthenia gravis were analyzed with multivariate logistic regression. We controlled for sex, age, low-income status, operation in a teaching hospital or not, preoperative coexisting medical conditions, and types of surgery and anesthesia. Multivariate logistic regressions were also used to assess the impact of preoperative myasthenia gravis-related emergency care and hospitalization on 30-day postoperative pneumonia, intensive care unit or prolonged stay, and increased medical expenditure. SAS version 9.1 (SAS Institute Inc., Cary, NC, USA) statistical software was used; two-sided *P* < 0.05 indicated significant differences between groups.

## Results

After matching procedure by propensity score (Table 1), the baseline characteristics showed no significant differences between surgical patients with and without myasthenia gravis in terms of age, sex, low income, operation in teaching hospital or not, type of surgery or of anesthesia, or preoperative coexisting mental disorders, hypertension, diabetes, chronic obstructive pulmonary diseases, hyperlipidemia, liver cirrhosis, congestive heart failure, and renal dialysis.

**Table 1:** Characteristics of surgical patients with and without myasthenia gravis.

	No MG (N = 22900)		MG (N = 2290)		
	n	(%)	n	(%)	*P*
Sex					0.7208
Female	14247	(62.2)	1416	(61.8)	
Male	8653	(37.8)	874	(38.2)	
Age, years					0.9894
20–29	2435	(10.6)	248	(10.8)	
30–39	3370	(14.7)	339	(14.8)	
40–49	4882	(21.3)	493	(21.5)	
50–59	4811	(21.0)	485	(21.2)	
60–69	3400	(14.9)	328	(14.3)	
≥70	4002	(17.5)	397	(17.3)	
Operation in teaching hospital	21539	(94.1)	2157	(94.2)	0.7937
Low income	575	(2.5)	65	(2.8)	0.3423
Coexisting medical conditions					
Mental disorders	4890	(21.4)	497	(21.7)	0.6975
Hypertension	4950	(21.6)	493	(21.5)	0.9229
Diabetes	2559	(11.2)	251	(11.0)	0.7565
COPD	2509	(11.0)	252	(11.0)	0.9441
Hyperlipidemia	1287	(5.6)	139	(6.1)	0.3745
Liver cirrhosis	371	(1.6)	41	(1.8)	0.5401
Congestive heart failure	334	(1.5)	34	(1.5)	0.9206
Renal dialysis	96	(0.4)	13	(0.6)	0.3020
Type of surgery					0.9996
Skin	429	(1.9)	43	(1.9)	
Breast	435	(1.9)	45	(2.0)	
Musculoskeletal	3666	(16.0)	362	(15.8)	
Respiratory	3596	(15.7)	363	(15.9)	
Cardiovascular	1432	(6.3)	144	(6.3)	
Digestive	3063	(13.4)	308	(13.5)	
Kidney, ureter, bladder	1377	(6.0)	132	(5.8)	
Delivery, CS, abortion	1186	(5.2)	123	(5.4)	
Neurosurgery	1820	(8.0)	179	(7.8)	
Eye	502	(2.2)	56	(2.5)	
Other	5394	(23.6)	535	(23.4)	
Type of anesthesia					0.7590
General	17595	(76.8)	1753	(76.6)	
Epidural or spinal	5305	(23.2)	537	(23.4)	

COPD = chronic obstructive pulmonary disease; MG = myasthenia gravis.

Compared with non-myasthenia gravis surgical patients (Table 2), patients with myasthenia gravis showed higher risks of postoperative complications, including pneumonia (OR = 2.09; 95% CI: 1.65–2.65), septicemia (OR = 1.31; 95% CI: 1.05–1.64), postoperative bleeding (OR = 1.71; 95% CI: 1.07–2.72), and overall complications (OR = 1.70; 95% CI: 1.44–2.00). Postoperative stay in intensive care unit (OR = 5.86; 95% CI: 5.23–6.57), prolonged stay, and increased medical expenditure were all significantly associated with preoperative myasthenia gravis.

**Table 2:** Risk of postoperative adverse events in association with myasthenia gravis in the multiple logistic regression models.

	No MG, %	MG, %	OR	(95% CI)^a^
Postoperative complications				
Pneumonia	2.1	4.1	2.09	(1.65–2.65)
Postoperative bleeding	0.6	0.9	1.71	(1.07–2.72)
Septicemia	3.2	4.0	1.31	(1.05–1.64)
Pulmonary embolism	0.1	0.1	1.18	(0.36–3.91)
Urinary tract infection	3.5	3.5	1.02	(0.80–1.30)
Stroke	2.3	1.9	0.80	(0.58–1.11)
Deep wound infection	0.4	0.3	0.73	(0.32–1.67)
Acute renal failure	0.9	0.5	0.58	(0.32–1.05)
Acute myocardial infarction	0.4	0.1	0.35	(0.11–1.12)
Any adverse events^c^	5.3	8.3	1.70	(1.44–2.00)
30-day in-hospital mortality	0.6	0.4	0.60	(0.29–1.24)
Intensive care unit stay	13.8	36.3	5.86	(5.23–6.57)
Length of hospital stay, days^b^	9.1±13.6	13.3±16.2	*p*<0.0001	
Medical expenditure, US$^b^	2060±3922	3338±5529	*p*<0.0001	

CI = confidence interval; ICU = intensive care unit; MG = myasthenia gravis; OR = odds ratio.

^a^Adjusted for all covariates listed in Table 1.

^b^Mean±SD

^c^Adverse events include with pneumonia, septicemia, postoperative bleeding.

Table 3 shows that the specific associations between myasthenia gravis and postoperative adverse events were significant in males (OR = 1.53; 95% CI: 1.18–1.97), females (OR = 1.86; 95% CI: 1.50–2.31), and every age group. These included 20–39 years (OR = 2.19; 95% CI: 1.48–3.25), 40–49 years (OR = 1.95; 95% CI: 1.29–2.97), 50–59 years (OR = 2.07; 95% CI: 1.47–2.92), and ≥60 years (OR = 1.36; 95% CI: 1.06–1.74). Myasthenia gravis was also associated with postoperative adverse events in people with no medical conditions (OR = 2.15; 95% CI: 1.67–2.78), 1 (OR = 1.38; 95% CI: 1.03–1.85) or more than two (OR = 1.56; 95% CI: 1.13–2.16). Compared with surgical patients without myasthenia gravis (Table 4), those with significant increased risk of postoperative adverse events included those with preoperative emergency care visits (OR = 2.89; 95% CI: 1.99–4.21), hospitalization (OR = 2.79; 95% CI: 2.20–3.52), thymectomy (OR = 3.71; 95% CI: 1.23–11.2), and high medical expenditure (OR = 2.77; 95% CI: 2.08–3.67). The highest risk of postoperative adverse events was found in low-income patients with myasthenia gravis (OR = 5.98; 95% CI: 3.12–11.5). Compared with patients without myasthenia gravis, the ORs of postoperative adverse events for patients with myasthenia gravis who had symptomatic therapy, chronic immunotherapy, and short-term immunotherapy were 1.76 (95% CI 1.50–2.08), 1.70 (95% CI 1.36–2.11), and 4.36 (95% CI 2.11–9.04), respectively.

**Table 3:** Stratification analysis of postoperative adverse events between myasthenia gravis patients and control by age, sex, and coexisting medical conditions.

			Postoperative adverse events^a^			
		n	Events	Incidence, %	OR	(95% CI)^b^
Female	No MG	14247	654	4.6	1.00	(reference)
	MG	1416	111	7.8	1.86	(1.50–2.31)
Male	No MG	8653	561	6.5	1.00	(reference)
	MG	874	79	9.0	1.53	(1.18–1.97)
20–39 years	No MG	5805	154	2.7	1.00	(reference)
	MG	587	33	5.6	2.19	(1.48–3.25)
40–49 years	No MG	4882	155	3.2	1.00	(reference)
	MG	493	29	5.9	1.95	(1.29–2.97)
50–59 years	No MG	4811	227	4.7	1.00	(reference)
	MG	485	44	9.1	2.07	(1.47–2.92)
≥60 years	No MG	7402	679	9.2	1.00	(reference)
	MG	725	84	11.6	1.36	(1.06–1.74)
0 medical conditions	No MG	11426	403	3.5	1.00	(reference)
	MG	1143	80	7.0	2.15	(1.67–2.78)
1 medical conditions	No MG	7285	455	6.3	1.00	(reference)
	MG	721	59	8.2	1.38	(1.03–1.85)
≥2 medical conditions	No MG	4189	357	8.5	1.00	(reference)
	MG	426	51	12.0	1.56	(1.13–2.16)

CI = confidence interval; MG = myasthenia gravis; OR = odds ratio.

^a^Postoperative adverse events included with pneumonia, septicemia, postoperative bleeding.

^b^Adjusted for all covariates listed in Table 1.

**Table 4:** Risks of postoperative adverse events associated with preoperative characteristics of myasthenia gravis.

		Postoperative adverse events^a^			
Preoperative characteristics	n	Events	Incidence, %	OR	(95% CI)^b^
No MG	22900	1215	5.3	1.00	(reference)
MG with symptomatic therapy	1658	141	8.5	1.76	(1.50–2.08)
MG with chronic immunotherapy	1333	117	8.8	1.70	(1.36–2.11)
MG with short-term immunotherapy	77	16	20.8	4.36	(2.11–9.04)
Emergency care for MG					
MG without emergency	2042	153	7.5	1.55	(1.29–1.85)
MG with emergency	248	37	14.9	2.89	(1.99–4.21)
Hospitalization for MG					
MG without hospitalization	1561	94	6.0	1.21	(0.97–1.51)
MG with hospitalization	729	96	13.2	2.79	(2.20–3.52)
Thymectomy					
MG without thymectomy	2259	186	8.2	1.68	(1.42–1.98)
MG with thymectomy	31	4	12.9	3.71	(1.23–11.2)
Low income					
MG without low income	2225	177	8.0	1.61	(1.36–1.91)
MG with low income	65	13	20.0	5.98	(3.12–11.5)
Medical expenditure of MG^c^					
Low	856	54	6.3	1.24	(0.93–1.66)
Moderate	956	71	7.4	1.58	(1.22–2.04)
High	478	65	13.6	2.77	(2.08–3.67)

CI = confidence interval; MG = myasthenia gravis; OR = odds ratio.

^a^Adverse events include pneumonia, septicemia, postoperative bleeding.

^b^Adjusted for all covariates listed in Table 1.

^c^Dollars/days

## Discussion

This nationwide retrospective population-based study shows myasthenia gravis as an independent risk factor increasing postoperative adverse outcomes after major surgery. Risks of pneumonia, septicemia, postoperative bleeding, and intensive care unit admission were found higher in those with myasthenia gravis, although 30-day in-hospital mortality did not increase. Prolonged length of stay and increased medical expenditure after surgery were also noted in patients with myasthenia gravis. Women, patients under age 60, and those without other medical conditions had higher incidences of postoperative adverse events. We also provided some parameters which could be easily obtained from personal history that could be helpful in predicting risks of postoperative adverse events. Unlike other investigations [7–11], this study is unique in its large sample size, selection of control group by matching procedure with propensity score and multivariate adjustment for potential confounding factors.

As with other systemic autoimmune diseases, myasthenia gravis often appears with other autoimmune diseases such as thyroid disease, polymyositis/dermatomyositis, systemic lupus erythematosus, rheumatoid arthritis, cardiomyositis, subclinical heart dysfunction, and cancer [20,21,22]. Although these comorbidities increase the complexity of medical treatment, advances in medical science have reduced mortality from myasthenia gravis [20,23].

Some previous studies suggested that surgical patients’ postoperative adverse events could be influenced by age, sex, low income, hospital type, types of surgery and of anesthesia, and coexisting medical conditions (mental disorders, diabetes, chronic obstructive pulmonary disease, liver cirrhosis, congestive heart failure, and renal dialysis) [14–17]. These sociodemographics, coexisting medical conditions, and operational characteristics were considered as potential confounding factors when investigating associations between myasthenia gravis and postoperative adverse events. In order to reduce such confounding effects, we used propensity score-matching procedure to adjust the preoperative difference between patients with and without myasthenia gravis. The further multiple logistic regressions were used to adjust residual confounding bias. The increased risk of postoperative adverse events in patients with myasthenia gravis was significant in males, females, every age group, and people with and without medical conditions. From an epidemiological viewpoint, this strengthens the association between myasthenia gravis and postoperative adverse events.

The findings of increased postoperative pneumonia, bleeding, and infection in patients with myasthenia gravis may have several possible reasons. First, the pharmacologic effects of immunosuppressant medications such as steroids, azathioprine, cyclophosphamide and mycophenolate mofetil put myasthenia gravis patients into immunocompromised status so they suffer from more opportunistic infections [24]. Steroids have been linked to increased reintervention rates for post-tonsillectomy bleeding [25]. Azathioprine and cyclophosphamide may also play a role in bleeding episodes due to myelotoxicity [26,27]. Second, empirical delay in extubation after surgery may cause unavoidable atelectasis [28,29], delay ambulation, and prolong intensive care unit stay, which might increase the risk of ventilator-associated pneumonia and embolism [30]. Third, the systemic inflammatory course of myasthenia gravis with other autoimmune comorbidities might directly contribute to systemic adverse events [21,22]. Fourth, poor communication between intubated patients and staff as well as mental illness-related communication issues might slow detection of adverse events.

Age and gender impacts on myasthenia gravis mortality have been controversial [22,23,31,32]. In our study, we found the incidence of postoperative complications lower in male and elderly patients than in females and other age groups. Similarly, a 2015 nationwide population-based study in Denmark enrolled 702 AChR-Ab-seropositive myasthenia gravis patients and found myasthenia gravis mortality rates were higher in women and early-onset myasthenia gravis, perhaps due to increased frequency of combined autoimmune disorders and thymoma in these patients [22,31,32]. Although mortality and postoperative complications differ, these groups of patients could be followed with greater care after surgery. Interestingly, the incidence of postoperative adverse events was higher in patients without medical conditions than in the other two groups. This could be because fewer comorbidities in early-onset myasthenia gravis patients still yield higher mortality [31], or other coexisting medical conditions’ impacts being eliminated by propensity score-matching procedure.

Another striking finding of our study is that myasthenia gravis patients had positive correlation with postoperative adverse events after most coexisting medical conditions were adjusted in the areas of low income, preoperative emergency care visit, hospitalization, and thymectomy. The myasthenia crisis could be provoked by other systemic issues that require emergency medical assistance [33]. Although patients with MG have a greater morbidity then general population, thymectomy was found to be a safe procedure [7]. As the condition worsens toward acute exacerbation, a treatment course with immunoglobulin G or plasmaphoresis and other intensive supportive care usually takes 2 to 5 days in hospital [32]. Based on this sequence, the severity of patient disease could be deduced from patient receiving emergency care or being hospitalized. Two recent studies found that unstable myasthenia gravis before surgery, preoperative plasmapheresis, and patients with a history of myasthenic crisis have increased incidence of myasthenic crisis after surgery [34,35]. Patients with these histories might be considered more vulnerable, and this might explain the significant increase in postoperative adverse events. Thymoma has been found in 10–15% of myasthenia gravis patients, mostly in early-onset and generalized forms of the disease [36,37]. Thymoma-associated myasthenia gravis has been associated with haematological autoimmune disorder, severe cardiomyositis, heart conduction abnormalities, and cancers [32]; it should be considered a paraneoplastic disease whose complex comorbidity interactions can contribute to postoperative adverse effects.

Our study has several limitations, starting with the lack of detailed patient lifestyle information (smoking, alcohol drinking, and physical activity) as well as lack of physical, medical, and laboratory examinations in the retrospective medical claims data we used. Second, we used ICD-9-CM codes claimed by physicians for myasthenia gravis without clarifying the severity of disease. Future researchers can use means such as Myasthenia Gravis Foundation of America Clinical Classification to assure comparability of investigations [38]. The influence of disease severity on postoperative complications can be only evaluated by indirect parameters such as history of emergency care and hospital admission. In addition, the coexisting medical conditions and postoperative complications identified using insurance claims data might represent an underestimate, because some patients with very minor illness might not seek medical treatment. However, this condition may distribute equally between surgical patients with and without myasthenia gravis. Finally, although we used propensity score matching procedure and multivariate logistic regressions to eliminate bias from confounders, residual confounding is always possible.

## Conclusion

Our study found increased postoperative pneumonia, septicemia, bleeding, stay in intensive care unit, prolonged stay, and increased medical expenditure for patients with myasthenia gravis receiving major surgery compared with control. In particular, postoperative adverse events significantly increased in myasthenia gravis patients with preoperative history of hospitalization, emergency visit, thymectomy, low income and high medical expenditure. These findings have important clinical implications, both for their predictive value and for showing the urgent need to improve management of surgical patients with myasthenia gravis.

We suggest comprehensive perioperative assessment and greater understanding of the complex issues of care for patients with myasthenia gravis who undergo major surgery.

## Abbreviations

CI: confidence interval
ICD-9-CM: International Classification of Diseases, Ninth Revision, Clinical Modification
OR: odds ratio

## References

[1] Spillane J, Higham E, Kullmann DM. Myasthenia gravis. BMJ. 2012;345: e8497 doi: 10.1136/bmj.e8497 23261848 10.1136/bmj.e8497

[2] Carr AS, Cardwell CR, McCarron PO, McConville J. A systematic review of population-based epidemiological studies in myasthenia gravis. BMC Neurol. 2010;10: 46 doi: 10.1186/1471-2377-10-46 20565885 10.1186/1471-2377-10-46 PMC2905354

[3] Guptill JT, Sharma BK, Marano A, Soucy A, Krueger A, Sanders DB. Estimated cost of treating myasthenia gravis in an insured U.S. population. Muscle Nerve. 2012;45: 363–366. doi: 10.1002/mus.22327 22334170 10.1002/mus.22327

[4] Kulaksizoglu IB. Mood and anxiety disorders in patients with myasthenia gravis aetiology, diagnosis and treatment. CNS Drugs. 2007;21: 473–481. 17521227 10.2165/00023210-200721060-00004

[5] Martínez-Lapiscina EH, Erro ME, Ayuso T, Jericó I. Myasthenia gravis: sleep quality, quality of life, and disease severity. Muscle Nerve. 2012;46: 174–180. doi: 10.1002/mus.23296 22806365 10.1002/mus.23296

[6] Harvey AM, Lilienthal JL, Talbot SA. Observations on the nature of myasthenia gravis: the effect of thymectomy on neuro-muscular transmission. J Clin Invest. 1942;21: 579–588. doi: 10.1172/JCI101336 16694948 10.1172/JCI101336 PMC435176

[7] Alshaikh JT, Amdur R, Sidawy A, Trachiotis G, Kaminski HJ. Thymectomy is safe for myasthenia gravis patients: Analysis of the NSQIP database. Muscle Nerve. 2016;53: 370–374. doi: 10.1002/mus.24904 26355385 10.1002/mus.24904

[8] Cheng C, Liu Z, Xu F, Deng Z, Feng H, Lei Y, et al Clinical outcome of juvenile myasthenia gravis after extended transsternal thymectomy in a Chinese cohort. Ann Thorac Surg. 2013;95: 1035–1041. doi: 10.1016/j.athoracsur.2012.11.074 23374447 10.1016/j.athoracsur.2012.11.074

[9] Marulli G, Schiavon M, Perissinotto E, Bugana A, Di Chiara F, Rebusso A, et al Surgical and neurologic outcomes after robotic thymectomy in 100 consecutive patients with myasthenia gravis. J Thorac Cardiovasc Surg. 2013;145: 730–735. doi: 10.1016/j.jtcvs.2012.12.031 23312969 10.1016/j.jtcvs.2012.12.031

[10] Tomulescu V, Sgarbura O, Stanescu C, Valciu C, Campeanu A, Herlea V, et al Ten-year results of thoracoscopic unilateral extended thymectomy performed in nonthymomatous myasthenia gravis. Ann Surg. 2011;254: 761–765. doi: 10.1097/SLA.0b013e31823686f6 22005151 10.1097/SLA.0b013e31823686f6

[11] Keijzers M, de Baets M, Hochstenbag M, Abdul-Hamid M, Zur Hausen A, van der Linden M, et al Robotic thymectomy in patients with myasthenia gravis: neurological and surgical outcomes. Eur J Cardiothorac Surg. 2015;48: 40–45. doi: 10.1093/ejcts/ezu352 25234092 10.1093/ejcts/ezu352

[12] Pakzad Z, Aziz T, Oger J. Increasing incidence of myasthenia gravis among elderly in British Columbia, Canada. Neurology. 2011;76: 1526–1528. doi: 10.1212/WNL.0b013e318217e735 21519005 10.1212/WNL.0b013e318217e735

[13] Buzello W, Noeldge G, Krieg N, Brobmann GF. Vecuronium for muscle relaxation in patients with myasthenia gravis. Anesthesiology. 1986;64: 507–509. 2870666 10.1097/00000542-198604000-00017

[14] Yeh CC, Liao CC, Chang YC, Jeng LB, Yang HR, Shih CC, et al Adverse outcomes after noncardiac surgery in patients with diabetes: a nationwide population-based retrospective cohort study. Diabetes Care. 2013;36: 3216–3221. doi: 10.2337/dc13-0770 23990518 10.2337/dc13-0770 PMC3781492

[15] Liao CC, Shen WW, Chang CC, Chang H, Chen TL. Surgical adverse outcomes in patients with schizophrenia: a population-based study. Ann Surg. 2013;257: 433–438. doi: 10.1097/SLA.0b013e31827b9b25 23241870 10.1097/SLA.0b013e31827b9b25

[16] Ke CC, Lin CS, Yeh CC, Chung CL, Hung CJ, Liao CC, et al Adverse outcomes after non-chest surgeries in patients with pulmonary tuberculosis: a nationwide study. PLoS One. 2015;10: e0133064 doi: 10.1371/journal.pone.0133064 26172153 10.1371/journal.pone.0133064 PMC4501732

[17] Chou CL, Lee WR, Yeh CC, Shih CC, Chen TL, Liao CC. Adverse outcomes after major surgery in patients with pressure ulcer: a nationwide population-based retrospective cohort study. PLoS One. 2015;10: e0127731 doi: 10.1371/journal.pone.0127731 26000606 10.1371/journal.pone.0127731 PMC4441478

[18] Rosenbaum PR, Rubin DB. Constructing a control group using multivariate matched sampling methods that incorporate the propensity score. Am Stat. 1985;39: 33–38.

[19] Wijeysundera DN, Beattie WS, Austin PC, Hux JE, Laupacis A. Epidural anaesthesia and survival after intermediate-to-high-risk non-cardiac surgery: a population-based cohort study. Lancet. 2008;372: 562–569. doi: 10.1016/S0140-6736(08)61121-6 18692893 10.1016/S0140-6736(08)61121-6

[20] Andersen JB, Owe JF, Engeland A, Gilhus NE. Total drug treatment and comorbidity in myasthenia gravis: a population-based cohort study. Eur J Neurol. 2014;21: 948–955. doi: 10.1111/ene.12439 24712740 10.1111/ene.12439 PMC4238850

[21] Gilhus NE, Nacu A, Andersen JB, Owe JF. Myasthenia gravis and risks for comorbidity. Eur J Neurol. 2015;22: 17–23. doi: 10.1111/ene.12599 25354676 10.1111/ene.12599

[22] Fang F, Sveinsson O, Thormar G, Granqvist M, Askling J, Lundberg IE, et al The autoimmune spectrum of myasthenia gravis: a Swedish population-based study. J Intern Med. 2015;277: 594–604. doi: 10.1111/joim.12310 25251578 10.1111/joim.12310

[23] Alshekhlee A, Miles JD, Katirji B, Preston DC, Kaminski HJ. Incidence and mortality rates of myasthenia gravis and myasthenic crisis in US hospitals. Neurology. 2009;72: 1548–1554. doi: 10.1212/WNL.0b013e3181a41211 19414721 10.1212/WNL.0b013e3181a41211

[24] Termsarasab P, Katirji B. Opportunistic infections in myasthenia gravis treated with mycophenolate mofetil. J Neuroimmunol. 2012;249: 83–85. doi: 10.1016/j.jneuroim.2012.04.016 22613699 10.1016/j.jneuroim.2012.04.016

[25] Plante J, Turgeon AF, Zarychanski R, Lauzier F, Vigneault L, Moore L, et al Effect of systemic steroids on post-tonsillectomy bleeding and reinterventions: systematic review and meta-analysis of randomised controlled trials. BMJ. 2012;345: e5389 doi: 10.1136/bmj.e5389 22930703 10.1136/bmj.e5389 PMC3429364

[26] Connell WR, Kamm MA, Ritchie JK, Lennard-Jones JE. Bone marrow toxicity caused by azathioprine in inflammatory bowel disease: 27 years of experience. Gut. 1993;34: 1081–1085. 8174958 10.1136/gut.34.8.1081 PMC1374358

[27] Fraiser LH, Kanekal S, Kehrer JP. Cyclophosphamide toxicity: characterising and avoiding the problem. Drugs. 1991;42: 781–795. 1723374 10.2165/00003495-199142050-00005

[28] Duggan M, Kavanagh BP. Pulmonary atelectasis: a pathogenic perioperative entity. Anesthesiology. 2005;102: 838–854. 15791115 10.1097/00000542-200504000-00021

[29] Seneviratne J, Mandrekar J, Wijdicks EF, Rabinstein AA. Predictors of extubation failure in myasthenic crisis. Arch Neurol. 2008;65: 929–933. doi: 10.1001/archneur.65.7.929 18625860 10.1001/archneur.65.7.929

[30] Ramagopalan SV, Wotton CJ, Handel AE, Yeates D, Goldacre MJ. Risk of venous thromboembolism in people admitted to hospital with selected immune-mediated diseases: record-linkage study. BMC Med. 2011;9: 1 doi: 10.1186/1741-7015-9-1 21219637 10.1186/1741-7015-9-1 PMC3025873

[31] Hansen JS, Danielsen DH, Somnier FE, Frøslev T, Jakobsen J, Johnsen SP, et al Mortality in myasthenia gravis: a nationwide population-based follow-up study in Denmark. Muscle Nerve. 2016;53: 73–77. doi: 10.1002/mus.24697 25914186 10.1002/mus.24697

[32] Gilhus NE, Verschuuren JJ. Myasthenia gravis: subgroup classification and therapeutic strategies. Lancet Neurol. 2015;14: 1023–1036. doi: 10.1016/S1474-4422(15)00145-3 26376969 10.1016/S1474-4422(15)00145-3

[33] Ramos-Fransi A, Rojas-García R, Segovia S, Márquez-Infante C, Pardo J, Coll-Cantí J, et al Myasthenia gravis: descriptive analysis of life-threatening events in a recent nationwide registry. Eur J Neurol. 2015;22: 1056–1061. doi: 10.1111/ene.12703 25847221 10.1111/ene.12703

[34] Ando T, Omasa M, Kondo T, Yamada T, Sato M, Menju T, et al Predictive factors of myasthenic crisis after extended thymectomy for patients with myasthenia gravis. Eur J Cardiothorac Surg. 2015;48: 705–709. doi: 10.1093/ejcts/ezu530 25618314 10.1093/ejcts/ezu530

[35] Leuzzi G, Meacci E, Cusumano G, Cesario A, Chiappetta M, Dall'armi V, et al Thymectomy in myasthenia gravis: proposal for a predictive score of postoperative myasthenic crisis. Eur J Cardiothorac Surg. 2014;45: e76–e88. doi: 10.1093/ejcts/ezt641 24525106 10.1093/ejcts/ezt641

[36] Marx A, Pfister P, Schalke B, et al The different roles of the thymus in the pathogenesis of the various myasthenia gravis subtypes. Autoimmun Rev. 2013;12: 875–884. doi: 10.1016/j.autrev.2013.03.007 23535159 10.1016/j.autrev.2013.03.007

[37] Skeie GO, Apostolski S, Evoli A, Gilhus NE, Illa I, Harms L, et al Guidelines for treatment of autoimmune neuromuscular transmission disorders. Eur J Neurol. 2010;17: 893–902. doi: 10.1111/j.1468-1331.2010.03019.x 20402760 10.1111/j.1468-1331.2010.03019.x

[38] Jaretzki A 3rd, Barohn RJ, Ernstoff RM, Kaminski HJ, Keesey JC, Penn AS, Sanders DB. Myasthenia gravis: recommendations for clinical research standards. Task Force of the Medical Scientific Advisory Board of the Myasthenia Gravis Foundation of America. Neurology. 2000;55: 16–23. 10891897 10.1212/wnl.55.1.16

